# Comparative analysis of NBS-LRR genes and their response to *Aspergillus flavus* in *Arachis*

**DOI:** 10.1371/journal.pone.0171181

**Published:** 2017-02-03

**Authors:** Hui Song, Pengfei Wang, Changsheng Li, Suoyi Han, Chuanzhi Zhao, Han Xia, Yuping Bi, Baozhu Guo, Xinyou Zhang, Xingjun Wang

**Affiliations:** 1 Biotechnology Research Center, Shandong Academy of Agricultural Sciences; Shandong Provincial Key laboratory of Crop Genetic Improvement, Ecology and Physiology, Jinan, China; 2 College of Life Science, Shandong Normal University, Jinan, China; 3 Henan Academy of Agricultural Sciences, Zhengzhou, China; 4 Crop Protection and Management Research Unit, USDA-ARS, Tifton, Georgia, United States of America; Texas Tech University, UNITED STATES

## Abstract

Studies have demonstrated that nucleotide-binding site–leucine-rich repeat (NBS–LRR) genes respond to pathogen attack in plants. Characterization of NBS–LRR genes in peanut is not well documented. The newly released whole genome sequences of *Arachis duranensis* and *Arachis ipaënsis* have allowed a global analysis of this important gene family in peanut to be conducted. In this study, we identified 393 (AdNBS) and 437 (AiNBS) NBS–LRR genes from *A*. *duranensis* and *A*. *ipaënsis*, respectively, using bioinformatics approaches. Full-length sequences of 278 AdNBS and 303 AiNBS were identified. Fifty-one orthologous, four AdNBS paralogous, and six AiNBS paralogous gene pairs were predicted. All paralogous gene pairs were located in the same chromosomes, indicating that tandem duplication was the most likely mechanism forming these paralogs. The paralogs mainly underwent purifying selection, but most LRR 8 domains underwent positive selection. More gene clusters were found in *A*. *ipaënsis* than in *A*. *duranensis*, possibly owing to tandem duplication events occurring more frequently in *A*. *ipaënsis*. The expression profile of NBS–LRR genes was different between *A*. *duranensis* and *A*. *hypogaea* after *Aspergillus flavus* infection. The up-regulated expression of NBS–LRR in *A*. *duranensis* was continuous, while these genes responded to the pathogen temporally in *A*. *hypogaea*.

## Introduction

In the environment, plants face attacks from pathogens and pests. Plants have evolved innate immunity systems against these challenges. The innate immunity system has been classified into pattern-triggered immunity (PTI) and effector-triggered immunity (ETI) [1]. PTI is mediated by surface-localized pattern recognition receptors (PRRs) that recognize pathogen-associated molecular patterns (PAMPs). ETI is mediated by intracellular immune receptors and directly or indirectly depends on resistance genes (*R* genes). *R* genes can be divided into at least five groups. The biggest group is nucleotide-binding site–leucine-rich repeat (NBS–LRR) genes [2].

NBS–LRR genes are distributed widely in plants. Researchers have studied this gene family in many plant genomes, including *Arabidopsis thaliana* [3], *Glycine max* [4], *Lotus japonicus* [5], *Medicago truncatula* [6], *Oryza sativa* [7], and *Triticum aestivum* [8]. NBS–LRR genes can be classified into two types (non-TIR and TIR) based on the N-terminal coiled-coil (CC) domain or a toll/mammalian interleukin-1 receptor (TIR) [3]. CC–NBS–LRR (CNL) genes are widely distributed in monocots and dicots but TIR–NBS–LRR (TNL) genes are mainly found in dicots, indicating that CNL genes originated before the divergence of monocots and dicots [9]. However, some studies have suggested that TNL genes actually originated earlier than CNL genes, and TNL genes were lost in grass and other species [10,11] because there were fewer TNL genes than CNL genes 100 million years ago, which only began to expand thereafter [11]. Recently, the RPW8 (resistance to powdery mildew 8)–NBS–LRR (RNL) gene was found to be an ancient NBS member that had a sister relationship with CNL genes in plants. However, the phylogenetic position of RNL genes has not been clearly determined [11].

*RFO1*, *RPW8*, and *WRR4*, three NBS–LRR genes from *Arabidopsis*, conferred resistance against *Fusarium* and powdery mildew fungi [12,13]. Heterologous expression of *Arabidopsis WRR4* in *Brassica* improved the resistance of transgenic lines to *Albugo candida* [14]. The function of at least 350 NBS–LRR genes was studied in rice [15–19]. Results showed that rice NBS–LRR genes played a crucial role in blast resistance. Moreover, overexpression of *M*. *truncatula RCT1* (TNL gene) in *Medicago sativa* could confer broad-spectrum resistance to anthracnose [20]. The expression of a cultivated peanut CNL gene was increased upon *Aspergillus flavus* infection, suggesting its roles in disease resistance [21].

Peanut (*Arachis hypogaea* L.), an important food and oil crop, is grown throughout the tropics and subtropics. Cultivated peanut is an allotetraploid (AABB genome) [22]. Its ancestral species are most likely the diploid *Arachis duranensis* and *Arachis ipaënsis*, which contributed the A and B subgenomes, respectively [23–26]. Previous studies showed that disease resistance of wild peanut was higher than that of cultivated peanut [27–29]. *A*. *flavus* can infect cultivated peanut before and after harvest [30] and produces carcinogenic mycotoxins, known as aflatoxins, which are toxic to both animal and human. Some peanut germplasms from China showed high resistance to *Aspergillus* colonization [31]. Identification and characterization of genes from wild or cultivated peanut for resistance to *A*. *flavus* is important for peanut breeding. The released whole genome sequences of *A*. *duranensis* and *A*. *ipaënsis* [32] allowed for systematic analysis of NBS–LRR genes in peanut. In this study, we identified NBS–LRR genes from *A*. *duranensis* and *A*. *ipaënsis* genomes using a bioinformatics approach. The chromosomal location, gene clusters, and phylogenetic relationships of these genes were analyzed. The expression of NBS–LRR genes in *A*. *duranensis* and cultivated peanut (Luhua 14) was analyzed after *A*. *flavus* infection.

## Materials and methods

### Sequence retrieval

The genome sequences of *A*. *duranensis* and *A*. *ipaënsis* have been released (http://peanutbase.org) [32]. The hidden Markov model (HMM) profile of the NB–APAF-1, R proteins, and CED-4 (ARC) domain (PF00931) was downloaded from the Pfam database (http://pfam.janelia.org). NBS–LRR proteins from two wild peanut were extracted using HMMER [33] and in-house Perl script. TIR, NBS, and LRR domains were confirmed in the Pfam database. The CC domain was surveyed using Paircoil2 (http://groups.csail.mit.edu/cb/paircoil2/). The *P*-score cutoff was 0.03.

### Phylogenetic relationships

Multiple sequence alignment of CNL and TNL full-length proteins from *A*. *duranensis* and *A*. *ipaënsis* was performed using MAFFT 7.0 [34]. A phylogenetic tree was constructed by MEGA 6.0 [35] using maximum likelihood (ML) with the Jones-Taylor-Thornton model and neighbor-joining (NJ) based on 1,000 replicates. If two genes from different species were clustered in pairs in the phylogenetic tree, these genes were considered as orthologous genes; if two genes from one species were clustered in pairs in the phylogenetic tree, these genes were considered as paralogous genes [36,37].

Protein sequences were converted into the corresponding nucleotide sequences by PAL2NAL [38]. PAML 4.0 [39] was used to calculate the K_a_/K_s_ (nonsynonymous/synonymous) ratio. Generally, K_a_/K_s_ = 1, >1, and <1 indicate neutral, positive, and purifying selection, respectively.

### Chromosomal location

The chromosomal location of NBS–LRR genes in *A*. *duranensis* and *A*. *ipaënsis* was obtained from peanutbase (http://peanutbase.org/). The map was generated by Circos v0.69 [40].

### Gene selection and qRT-PCR primer design

We analyzed the gene expression profile of a cultivated peanut after *A*. *flavus* infection (unpublished data) and found that the expression of some NBS–LRR genes responded to *A*. *flavus* infection. Here, we selected six highly expressed NBS–LRR genes for qRT-PCR analysis.

We used the sum of *A*. *duranensis* and *A*. *ipaënsis* sequences as the cultivated peanut genome because the complete genome of cultivated peanut has not been sequenced, and the sum of these two diploid genome sizes is equal to the genome size of cultivated peanut [32,41]. We designed primers for amplification of the *A*. *duranensis* sequences and their orthologous genes in cultivated peanut. qRT-PCR primers were designed based on the *A*. *duranensis* genome sequence using Beacon Designer 8.0. Primer information is provided in S1 Table. The actin gene was used as a reference gene for quantification [42].

### Inoculation of *A*. *flavus*

The *A*. *flavus* inoculation method was described by Zhang et al. [30]. Briefly, mature peanut seeds were surface-sterilized and cultivated on moist filter paper at 28°C for three days. The germinated peanut seeds were inoculated by immersing them in an *A*. *flavus* suspension of approximately 3 × 10^7^ spores/ml. Seeds immersed in sterile distilled water were used as the control. Seeds were placed in Petri dishes at 28°C and were harvested 1, 3, 5, and 7 days after treatment.

### RNA isolation and gene expression analysis

Total RNA was extracted using the hexadecyltrimethylammonium bromide (CTAB) method [43]. Two micrograms of RNA were used to synthesize first-strand cDNAs using the Reverse Transcriptase M-MLV System (Takara, Dalian, China). qRT-PCR was performed using Fast Start Universal SYBR Green Master (ROX) with a 7500 real-time PCR machine (ABI). The reaction was carried out as follows: 30 s at 95°C for denaturation, followed by 40 cycles of 5 s at 95°C, and 30 s at 60°C. A melting curve analysis was performed at the end of the PCR run over a range of 55–99°C. Three technical replicates were performed. The ^ΔΔ^C_t_ method was used for quantification [44]. One-way annova test was performed to obtain *P* values using GenStat 18.0 (Lawes Agricultural Trust, Oxford, UK). If *P* < 0.05, we considered the NBS–LRR genes as differentially expressed genes.

## Results and discussion

### Identification of NBS–LRR proteins in two wild peanut species

A total of 393 and 437 NBS–LRR-coding protein sequences were identified in *A*. *duranensis* and *A*. *ipaënsis*, respectively. However, 113 and 125 sequences from *A*. *duranensis* and *A*. *ipaënsis*, respectively, were excluded in this study because these sequences contained partial NBS domains or partial sequences. Song et al. [5] demonstrated that incomplete NBS–LRR sequences used in analyses can lead to incorrect results. Among the full-length sequences, two AdNBS and nine AiNBS sequences were considered potential pseudogenes because they contained either a premature stop codon or a frameshift mutation. Ultimately, 278 AdNBS and 303 AiNBS sequences were used for analysis in this study, named AdNBS1 to AdNBS278 and AiNBS1 to AiNBS303 (S2 and S3 Tables). AdNBS and AiNBS sequences contained more than one TIR, CC, NBS, and LRR domains, and these domains were randomly distributed in the amino acid sequences. Four NBS domains and 12 LRR domains were detected in AdNBS196, while six NBS domains and 14 LRR domains were detected in AiNBS196 (S2 and S3 Tables). Overall, AdNBS, including 30 CNL with 37 CC domains and 83 TNL sequences, contained 102 TIR domains. In total, 16 amino acid sequences contained only the NBS domain, and 123 amino acid sequences contained both NBS and LRR domains (Table 1). The AiNBS (38 CNL type and 90 TNL type sequences) contained 50 CC and 106 TIR domains. Twelve NBS-type and 135 NBS–LRR-type sequences were predicted (Table 1). Many LRR domains were distributed in the *Arachis* genomes (Table 1). In NBS–LRR sequences, 84.59% and 86.80% contained LRR domains in *A*. *duranensis* and *A*. *ipaënsis*, respectively. About 91.43% NBS–LRR sequences in *M*. *truncatula* [6] and 71.77% NBS–LRR sequences in *L*. *japonicas* [5] had LRR domains. We found that AdNBS and AiNBS contained more LRR8 than LRR4, LRR3, LRR5, and LRR1. The LRR5 domain only appeared in CNL proteins (S2 and S3 Tables).

**Table 1:** Number of NBS-LRR genes in *A*. *duranensis* and *A*. *ipaënsis*.

Type	*A*. *duranensis*	*A*. *ipaënsis*
CC type	37	50
CC-NBS	7	12
CC-NBS-LRR	30	38
TIR type	102	106
TIR-NBS	19	16
TIR-NBS-LRR	83	90
NBS type	16	12
NBS-LRR type	123	135
Total	278	303

Note: NBS-LRR type indicates sequence only contains NBS and LRR domains. NBS-LRR gene(s) indicate(s) nucleotide-binding site–leucine-rich repeat gene(s).

Although the genome of cultivated peanut has not been released, several studies have focused on the analysis of cultivated peanut NBS–LRR genes because of their potential importance in disease resistance. Bertioli et al. [45] cloned 78 full-length NBS–LRR genes from cultivated peanut and four wild peanuts (*A*.*duranensis*, *A*. *cardenasii*, *A*. *stenosperma*, and *A*. *simpsonii*). A total of 234 NBS–LRR genes were identified by PCR amplification in cultivated peanut [46]. We used NBS–LRR genes from two wild peanuts to search the scaffolds of cultivated peanut using the local BLASTN program. The results showed that orthologous genes of wild peanut NBS–LRR genes could be detected in cultivated peanut (data not shown). The NBS–LRR genes in cultivated peanut covered all NBS–LRR genes in two wild peanuts. The results showed the number of NBS–LRR genes in cultivated peanut was at least 830 (393 AdNBS and 437 AiNBS).

### Tandem duplication led to the formation of NBS–LRR paralogous genes in *Arachis*

**Fig 1:**
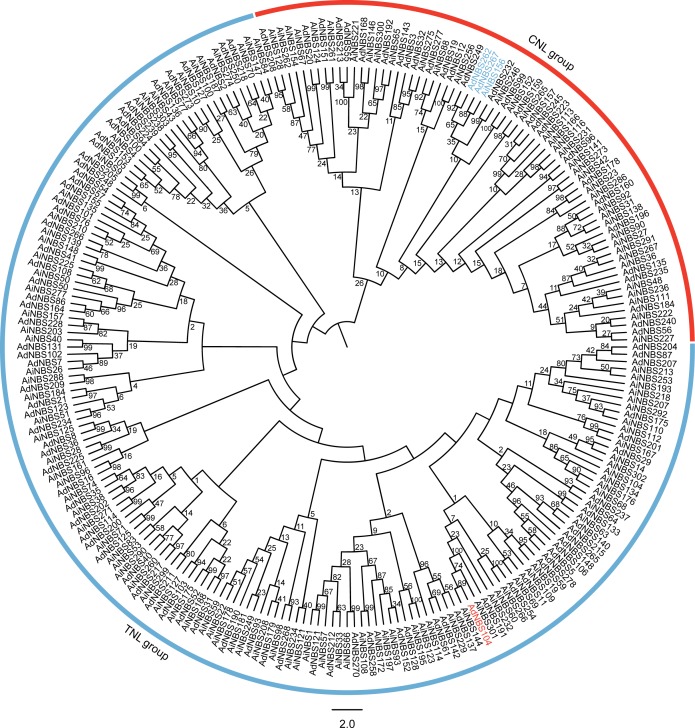
Phylogenetic tree of NBS-LRR from *A*. *duranensis* and *A*. *ipaënsis*. The phylogenetic tree was generated using CNL and TNL full-length proteins from *A*. *duranensis* and *A*. *ipaënsis* using MEGA 6.0 by the maximum likelihood (ML) with Jones-Taylor-Thornton model based on 1,000 bootstrap replicates.

NBS–LRR genes can be classified into two clades in phylogenetic trees, TNL and CNL groups [3]. The AdNBS and AiNBS phylogenetic tree also contained these two groups based on ML and NJ methods. However, one CNL sequence (AdNBS104) nested into the TNL group, and three TNL sequences (AdNBS262, AdNBS267, and AiNBS156) clustered together with CNL proteins (Fig 1 and S1 Fig). In *Eucalyptus grandis*, three CNL genes were located in the TNL group, and one TNL gene was found in the CNL group [47]. Similar results were found for *M*. *truncatula* [6] and *Vitis vinifera* [48] NBS–LRR sequences. Song and Nan [6] found that eight TNL genes were nested in the CNL group. Two CNL sequences were found to group with TNL proteins [48]. We hypothesize that recombination events occurred in the NBS domain. Innes et al. [49] found that recombination occurred between some NBS domains from CNL and TNL proteins.

**Fig 2:**
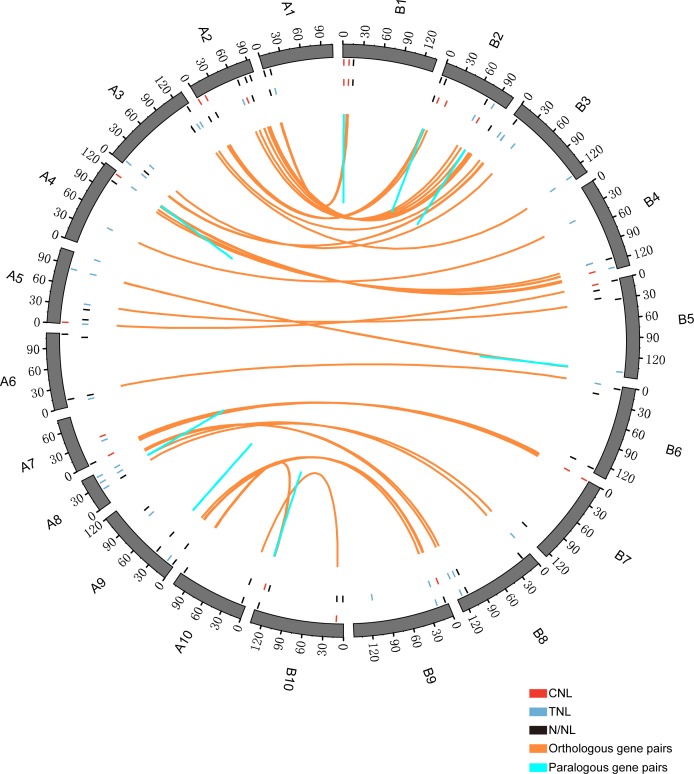
Chromosomal location and homologous gene relationship of NBS-LRR genes from *A*. *duranensis* and *A*. *ipaënsis*. The letters and numbers outside the circle represent species and chromosomes, respectively. A and B represent *A*. *duranensis* and *A*. *ipaënsis*, respectively.

We detected 51 orthologous gene pairs, four paralogous AdNBS gene pairs, and six paralogous AiNBS gene pairs based on both ML and NJ phylogenetic relationships (Fig 1, S1 Fig and S4 Table). Most of these 51 orthologous gene pairs were distributed in a similar locus on the corresponding chromosomes, except AdNBS2 (chromosome A2)—AiNBS274 (chromosome B3) (Fig 2 and S4 Table). Additionally, one gene pair contained both CNL (AdNBS104) and TNL (AiNBS144) genes, indicating that recombination was present between the CC and TIR domains. All of the paralogous gene pairs were located on one chromosome, indicating the tandem duplication is the main mechanism in forming NBS–LRR paralogs. Generally, tandem duplication produces novel resistant functions of NBS–LRR genes [50]. In soybean and *Medicago*, tandem duplication played a primary role in NBS–LRR gene expansion [4,6].

**Fig 3:**
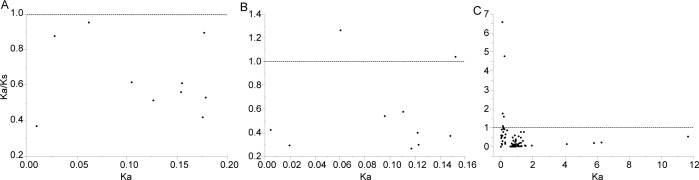
Comparison of K_a_/K_s_ values among NBS-LRR sequence, NBS and LRR regions. A, B and C represent NBS-LRR sequence, NBS and LRR regions, respectively.

Our results revealed that most paralogous genes and NBS and LRR protein-coding genes underwent purifying selection (Fig 3). LRR domains had significantly larger K_a_ values than the full-length protein (*P* < 0.01) and the NBS region (*P* < 0.01) in *A*. *duranensis* and *A*. *ipaënsis* (Fig 3), indicating faster evolution of protein sequences in LRR domains [51]. Most LRR 8 domains underwent positive selection, comparing to other type LRR domains (Fig 3). It is thought that rapidly evolving NBS–LRR genes have been under positive selection [15]. Therefore, LRR 8 exhibited signatures of rapid evolution in *Arachis*. Gu et al. [8] analyzed NBS–LRR proteins in bread wheat and found that 2.25% of LRR domains showed positive selection. Most likely the LRR domain co-evolved with pathogen effectors to mediate interaction directly or indirectly with pathogen molecules. The fact that most sites of positive selection were located on the surface of the folded protein may support this hypothesis [52,53].

### Gene cluster analysis in *A*. *duranensis* and *A*. *ipaënsis*

AdNBS and AiNBS genes were randomly distributed across 10 chromosomes. Six AdNBS genes were removed during cluster analysis because of lack of location information. Most AdNBS and AiNBS genes were located on chromosomes A2 and B2, respectively. The fewest AdNBS and AiNBS genes were found on chromosomes A6 and B7, respectively (Fig 2). CNL and TNL genes were found on each chromosome of *A*. *ipaënsis*, while CNL genes were absent on *A*. *duranensis* chromosome A8 and TNL genes were absent on chromosome A10.

**Fig 4:**
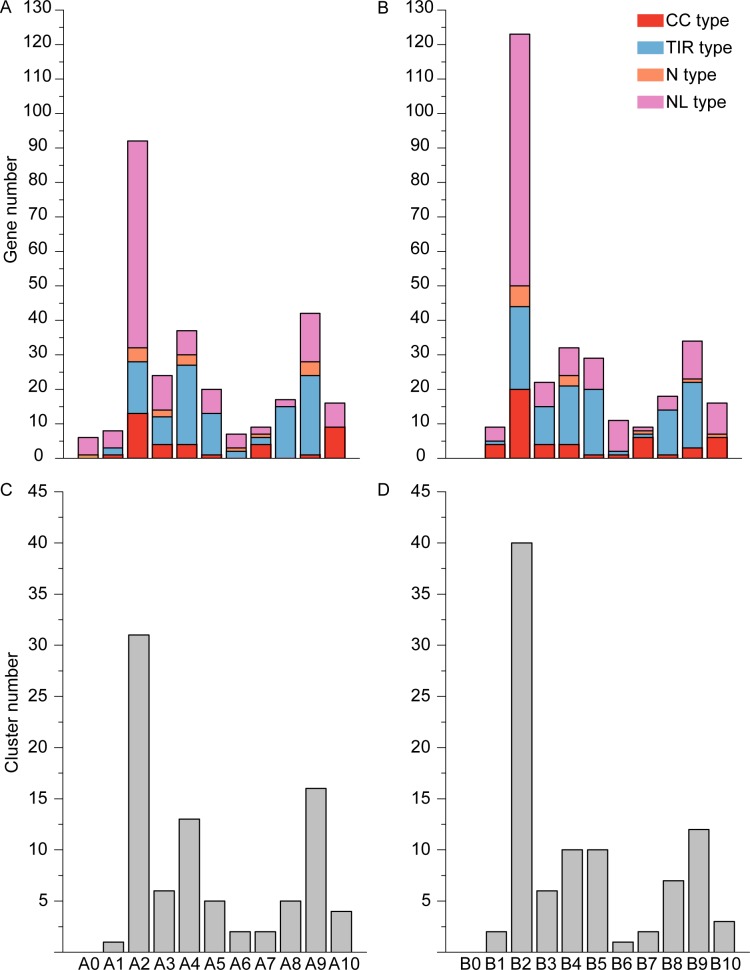
NBS-LRR gene number and cluster number in *A*. *duranensis* and *A*. *ipaënsis*. A and B represent gene number in *A*. *duranensis* and *A*. *ipaënsis*, respectively. C and D represent cluster number in *A*. *duranensis* and *A*. *ipaënsis*, respectively.

NBS–LRR gene clusters were reported in several legumes such as *G*. *max*, *L*. *japonicus*, *M*. *truncatula*, and *Phaseolus vulgaris* [4,6,54,55]. In this study, we defined a gene cluster as a chromosome region with two or more genes within 200 kb. A total of 85 and 93 clusters were detected in *A*. *duranensis* and *A*. *ipaënsis*, respectively. Chromosomes A2 and B2 contained the most clusters, while chromosomes A1 and B6 contained the fewest clusters (Fig 4). The number of clusters in *A*. *ipaënsis* is greater than that in *A*. *duranensis*, possibly because more tandem duplication events occurred in *A*. *ipaënsis*. About 57.14% and 84.62% of paralogous genes in *A*. *duranensis* and *A*. *ipaënsis*, respectively, were located within the clusters. Forming clusters of NBS–LRR genes appears to be a strategy for plants to quickly adapt to a changing spectrum of pathogens. In soybean, the *Rpg1* locus, containing NBS–LRR genes, played a role in resistance to *Pseudomonas syringae* [56]. *Rpsar-1*, a cluster of *R* genes in common bean, recognized *P*. *syringae* infection [57]. *MtQRR1*, containing a cluster of seven *R* genes, played an important role in *Ralstonia solanacearum* resistance in *M*. *truncatula* [58]. Kang et al. [4] found that clusters of NBS–LRR genes were highly correlated with many disease resistance QTLs in soybean.

### The expression of NBS–LRR genes under *A*. *flavus* infection

Previous studies showed that disease resistance of wild peanut was greater than that of cultivated peanut [27–29]. In this study, we examined the expression pattern of some NBS–LRR genes in *A*. *duranensis* and their orthologous genes in cultivated peanut. We did not select *A*. *ipaënsis* for expression analysis because we could not get seedlings from germination either in greenhouse or field conditions. High-throughput sequencing identified six NBS–LRR genes from cultivated peanut. These genes were highly induced upon *A*. *flavus* infection (unpublished data); but three NBS–LRR genes were selected for analysis by quantitative real-time PCR (qRT-PCR) because other genes cannot design primers.

**Fig 5:**
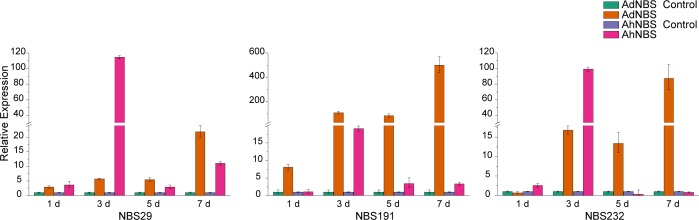
Expression of NBS-LRR genes from *A*. *duranensis* and *A*. *hypogaea* after *A*. *flavus* infection. The Y-axis indicates the relative expression level; X-axis indicates days of *A*. *flavus* infection. The standard errors are plotted using vertical lines.

qRT-PCR results showed that the expression of these genes was significantly induced by *A*. *flavus* infection (Fig 5). The expression profile of NBS–LRR genes was different between *A*. *duranensis* and *A*. *hypogaea* after *A*. *flavus* infection (Fig 5). The expression of NBS191 in *A*. *duranensis* was significantly higher than that in *A*. *hypogaea* at 1, 3, 5, and 7 d after inoculation (*P* < 0.01). The expression of NBS29 and NBS232 in *A*. *duranensis* was lower than that in *A*. *hypogaea* at 1 and 3 d (*P* < 0.01), while the expression in *A*. *duranensis* was significantly higher than that in *A*. *hypogaea* at 5 and 7 d (*P* < 0.01, Fig 5). It is important to note that the up-regulated expression of NBS–LRR in *A*. *duranensis* is continuous, while these genes respond to the pathogen temporally in *A*. *hypogaea*. The same result was found in *Arachis* lipoxygenase (*LOX*) genes [59]. *LOX* genes expression patterns differed significantly between wild-type peanut and cultivated peanut infected with *A*. *flavus* [59]. We speculated that polyploidization might be the reason for the reduced expression in cultivated peanut. Similar observations have been made in *Arabidopsis* and *Gossypium*. *Arabidopsis suecica* was hybrid of *A*. *thaliana* and *A*. *arenosa*. Wang et al. [60] found most genes in *A*. *thaliana* and *A*. *arenosa* were expressed at higher levels than in allotetraploids. In contrast, Flagel and Wendel [61] showed that the expression level of many genes was higher in allopolyploid *Gossypium* species than in a synthetic F1 hybrid. Transcriptome analysis showed that most genes were preferentially expressed in allotriploid *Populus* compared to their diploid parents [62].

Studies indicated that wild peanut is more resistant to diseases than cultivated peanut, and transferring resistance genes from wild species to cultivars could improve disease resistance of the cultivated peanut [22,29,63]. We speculated that cultivated peanut probably got both copies of resistance genes from two wild progenitors, but the expression of these genes might be modified in tetraploids. For example, epigenetic modifications, like DNA methylation, play important roles in regulation of gene expression. Investigating the mechanisms that control the differential expression of NBS–LRR genes in wild type and cultivated peanuts would be interesting. Global analysis of polyploidization induced genetic and epigenetic modifications may provide valuable clues for understanding the reprogramming of gene expression under biotic and abiotic stresses.

## Conclusion

In this study, we identified 278 AdNBS and 303 AiNBS full-length sequences. Most paralogous gene pairs were located on one chromosome, indicating tandem duplication was the main mechanism forming these paralogs. These paralogous genes mainly underwent purifying selection, while most LRR 8 domains underwent positive selection. More gene clusters were found in *A*. *ipaënsis* than in *A*. *duranensis*, possibly owing to more tandem duplication in *A*. *ipaënsis*. After *A*. *flavus* infection, NBS–LRR genes in *A*. *duranensis* responded more strongly and maintained a higher expression level compared to that in the cultivated peanut, which may provide clues for understanding differences in disease resistance between wild type and cultivated peanuts.

## Supporting information

S1 Fig Phylogenetic tree of NBS-LRR from *A*. *duranensis* and *A*. *ipaënsis*. The phylogenetic tree was generated using MEGA 6.0 by the neighbor-joining (NJ) method with 1,000 bootstrap replicates.
(TIF)

S1 Table qRT-PCR primers used in this study. (XLS)

S2 Table The information of NBS-LRR genes in *A*. *duranensis*. (XLS)

S3 Table The information of NBS-LRR genes in *A*. *ipaënsis*. (XLS)

S4 Table Homologous gene identification in *A*. *duranensis* and *A*. *ipaënsis*. (XLS)
