# Supplementary figures and images for: Comparative analysis of NBS-LRR genes and their response to Aspergillus flavus in Arachis
Source: PLoS One. 2017 Feb 3;12(2):e0171181. doi: 10.1371/journal.pone.0171181 (PMC5291535; doi:10.1371/journal.pone.0171181)

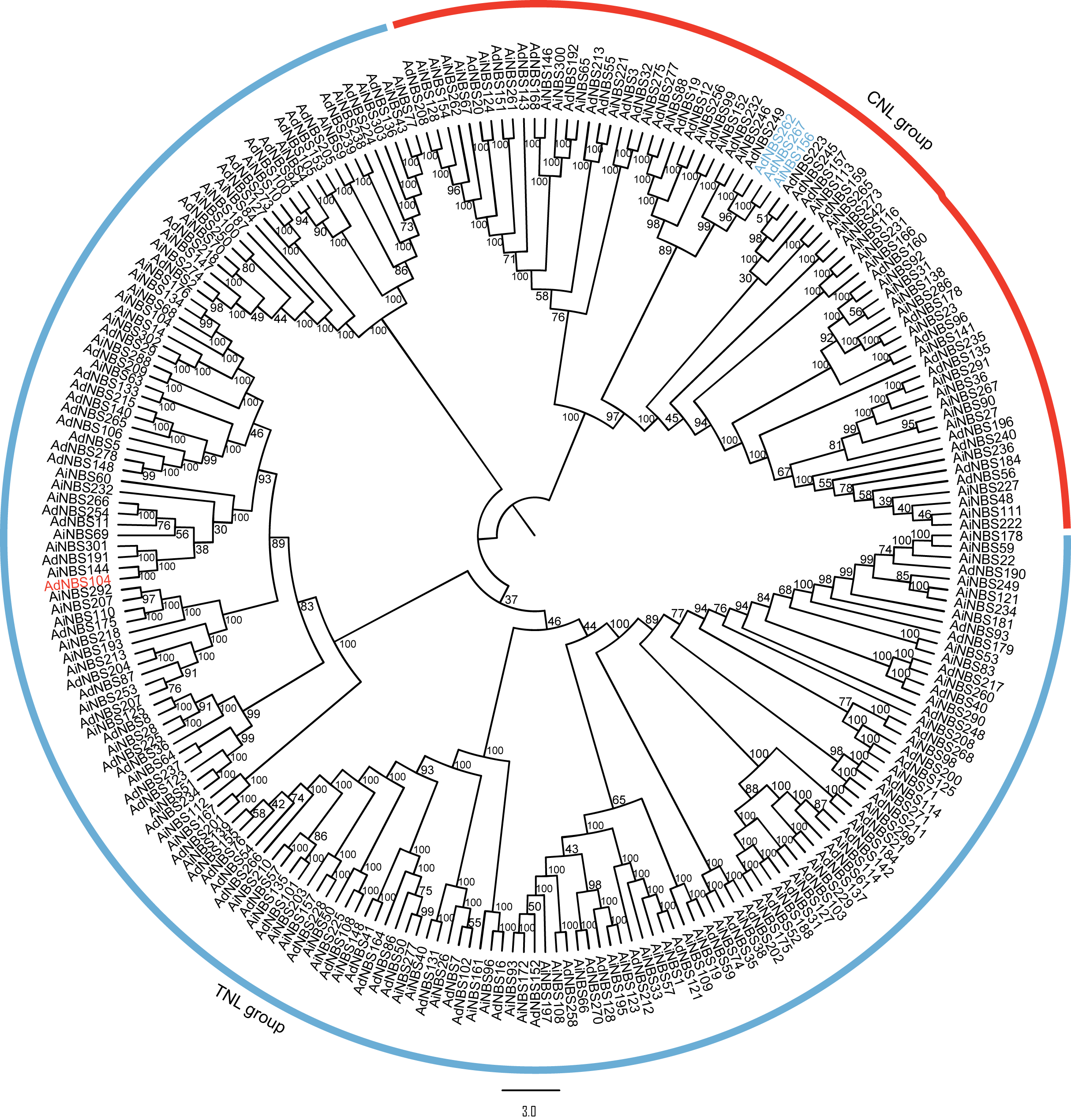

Supplement: S1 Fig — The phylogenetic tree was generated using MEGA 6.0 by the neighbor-joining (NJ) method with 1,000 bootstrap replicates. (TIF) [file pone.0171181.s001.tif]
